# Statins Do Not Influence Long-Term Rituximab Clinical Efficiency in Rheumatoid Arthritis Patients

**DOI:** 10.1155/2014/689426

**Published:** 2014-01-08

**Authors:** Diana Mazilu, Tania Gudu, Ruxandra Ionescu, Daniela Opris

**Affiliations:** ^1^Department of Rheumatology, “Sfanta Maria” Hospital, Blvd IonMihalache No. 37-39, 011172 Bucharest, Romania; ^2^“Carol Davila” University of Medicine, Bucharest, Romania

## Abstract

*Objective*. This longitudinal study aims to determine if statins inhibit the response to rituximab in rheumatoid arthritis (RA) patients. * Methods*. 41 patients initiating rituximab were included; 17 patients were exposed to the combination of statins and rituximab. The total cholesterol, erythrocyte sedimentation rate (ESR), and C-reactive protein (CRP) were assessed. The clinical response was evaluated using Disease Activity Score (DAS28) and European League against Rheumatism (EULAR) response at 6 and 18 months. *Results*. A tendency of increasing in DAS28 was observed in statin-exposed group but the correlation was very weak (at 18 months: *r* = 0.013, *P* = 0.952). The statin-exposed status was negatively and very weakly correlated with EULAR response at 6 months (*r* = −0.073, *P* = 0.661) and 18 months (*r* = −0.197, *P* = 0.244). There was a negative correlation between statin-exposed status and inflammatory markers values (ESR and CRP); however, the correlation was very weak. The use of statin did not influence the cardiovascular risk measured by modified Systematic Coronary Risk Evaluation (mSCORE). *Conclusions*. Long-term significant inhibitory effects of statins on rituximab treatment in RA have not been proved using clinical response scores or biologic markers.

## 1. Introduction

Rheumatoid arthritis (RA) is a chronic inflammatory joint disease and it should be regarded as a condition associated with a higher risk for cardiovascular diseases. Besides traditional risk factors (e.g., age, gender, smoking, blood pressure, and lipid levels), the increased risk appears to be strongly associated with the inflammatory burden [1].

Chronic inflammatory markers are independently associated with cardiovascular (CV) mortality and morbidity in RA patients [2–5]. The treatment goal is to achieve remission or low disease activity as soon as possible for every patient [6]. If synthetic disease-modifying antirheumatic drugs (DMARDs) fail, biologic agents should be prescribed in combination with methotrexate (MTX) or other DMARDs, since this combination has greater efficacy than monotherapy with most biological agents [6].

Rituximab is a chimeric anti-CD20 monoclonal antibody, which was approved in 1997 for treatment of indolent CD20, B-cell non-Hodgkin's lymphoma (NHL), and chronic lymphocytic leukemia and in 2006 for RA patients who have had an inadequate response to at least one tumor necrosis factor inhibitor alpha (TNF*α*-i) [7].

B-cell depletion is achieved through the apoptosis of B cell induced by the hypercrosslinking of CD20 molecules with rituximab. This process depends on the presence and integrity of lipid rafts in the cell membrane [8–10]. In vitro, statins interfere with the formation of cholesterol-rich microdomains within the plasma membrane—the lipid rafts, which impairs lymphoma cell killing by antibody-dependent cellular cytotoxicity and complement-dependent cytotoxicity by rituximab [8]. A small study [11] of patients with RA who have received rituximab plus statins (23 exposed patients) suggested that statins might decrease the response to rituximab. Another study [12] (26 exposed patients) showed no evidence of inferior clinical response in the first 6 months of treatment with rituximab in statin-exposed patients.

## 2. Patients and Methods

### 2.1. Study Design, Settings, and Participants

A sample of 74 patients from Sfanta Maria Hospital in Bucharest was included in this prospective cohort study which started in February 2010 and is still ongoing. All patients had high disease activity RA and failure to at least one anti-TNF and were switched to rituximab. The patients that discontinued the statin treatment or had associated statin after rituximab was initiated were excluded. A total number of 41 patients fulfilled the inclusion criteria. A consent was obtained from all participants before any examination test. The study was approved by the local Clinical Ethics Committee.

Rituximab was administrated intravenously, two 1 g infusions (given with 100 mg methylprednisolone), separated by an interval of two weeks. Treatment courses were repeated every 6 months for a period of 18 months (3 courses).

Anonymised datasets with baseline characteristics, including age, gender, disease duration, number of previous biologic DMARDS, anticyclic citrullinated peptide (anti-CCP) antibodies, and rheumatoid factor (RF) status of all patients were analyzed. Levels of ESR, CRP, total serum cholesterol, RF, and anti-CCP antibodies were determined at baseline and after 6, 12, and 18 months of treatment and local laboratory cut-off values were applied.

Disease activity markers were also provided (number of swollen and tender joints, Visual Analogue Scales, and DAS28). The EULAR response criteria at 6 months and 18 months were calculated and included in the analysis.

Since in Romania there is no national guideline for evaluating CV risk in patients with RA, we chose the modified SCORE (mSCORE) model, based on EULAR recommendations [1]. For that, the derived CV risk estimate SCORE was multiplied by 1.5 if at least two of the following criteria were present: disease duration of more than 10 years, RF and/or anti-CCP positivity, and the presence of severe extra-articular manifestations [1]. Romania is a country with high CV risk; consecutively we used the SCORE high risk chart for our study. mSCORE model was evaluated at baseline and after 18 months of treatment.

### 2.2. Statistical Analysis

Statistical analysis was performed using SPSS statistical software, version 20.0. The data were expressed as the mean ± SD. All statistical tests were two-sided and were performed at an *α* level of 0.05. The differences between groups were analyzed by Student's *t*-test or *χ* ^2^-test for categorical variables. Spearman's test was used for correlations. To describe the strength of the correlation, we used the Salkin scale for the absolute value of the correlation coefficient (*r*): very strong relationship (0.80 ≤ *r* ≤ 1.0), strong relationship (0.60 ≤ *r* < 0.80), moderate relationship (0.4 ≤ *r* < 0.6), weak relationship (0.2 ≤ *r* < 0.4), and very weak or no relationship (0.20 < *r* ≤ 0). The statistical significance was assumed for values of *P* < 0.05.

## 3. Results

Among 41 patients, 17 patients received statin treatment. The type of statin was chosen by attending physician. From the total number of statin-exposed patients, 10 patients (58.82%) were using rosuvastatin and 7 patients were simvastatin (41.17%). The statin dose was not taken into account, since an increase/decrease in statin dose after rituximab was initiated resulted in patients' exclusion from the study.

The baseline characteristics for all patients are summarized in Table 1. Statin-exposed patients were significantly older than unexposed patients (*P* = 0.018). DAS28 scores of both groups were similar at baseline (*P* = 0.993). mSCORE and other variables did not differ significantly at baseline.

Clinical outcome was evaluated using variables such as DAS28 and EULAR response, both measured at 6 and 18 months. Biological outcome was also assessed using inflammatory (ESR and CRP) and serological (RF) variables. There were no significant differences for patient characteristic at 6, 12, and 18 months.

Correlations between the statin-exposed status and these outcome variables were performed using Spearman's correlation test. The correlation was considered strong for a correlation coefficient *r* > 0.6 and statistical significance was assumed for values of *P* < 0.05.

**Figure 1:**
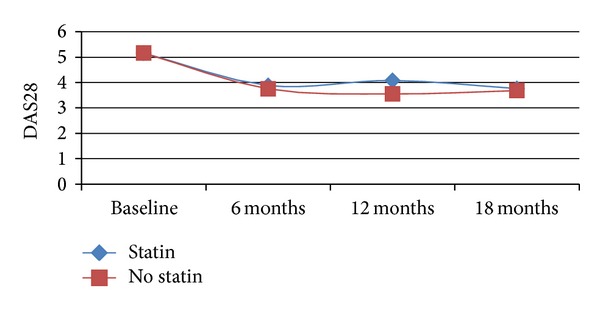
The influence of statins on course of disease activity (DAS28) over time.

DAS28 was identical for both groups at baseline. A tendency of increasing in DAS28 score can be observed in the statin-exposed group, as expressed in Figure 1. But statistical tests (Student's *t*-test) showed no significant difference for DAS28 score at 6, 12, and 18 months (*P* = 0.777, *P* = 0.303, and *P* = 0.136).

There was a very weak correlation between the use of statin and the clinical outcome expressed as DAS28 at 6 months (*r* = 0.077, *P* = 0.652) and DAS28 at 18 months (*r* = 0.013, *P* = 0.952).

**Figure 2:**
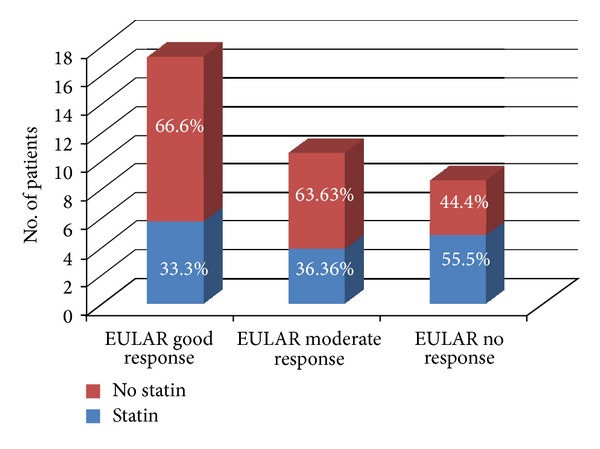
Effect of concomitant statins on clinical response at 18 months of treatment.

Patients with a good EULAR response at 18 months were fewer in the statin-exposed group, 6 (33.33%), compared to the nonexposed ones, 12 (66.66%) (Figure 2). It seems to be a tendency in a decreased EULAR response for those using a statin. But these results are not statistically significant.

The statin-exposed status was negatively correlated with EULAR response at 6 months (*r* = −0.073, *P* = 0.661) and 18 months (*r* = −0.197, *P* = 0.244). This could suggest that statin may inhibit rituximab effect, but the correlation was very weak and with no statistical significance.

We also assessed the CV risk using the mSCORE model at baseline and after 18 months. All patients met the criteria for applying a 1.5 multiplication factor, according to EULAR recommendations [1]. During treatment period, no severe CV events were reported. mSCORE of both groups was similar at baseline (*P* = 0.789) and after 18 months (*P* = 0.927) and was very weakly correlated with the use of statin at baseline (*r* = 0.133, *P* = 0.413) and after 18 months (*r* = 0.191, *P* = 0.239). This result suggests that the use of statin did not improve the CV risk for the patients included in the study.

As for the inflammatory markers, the correlation between the statin status and ESR and CRP were as follows: at 6 months: CRP (*r* = −0.126, *P* = 0.434), ESR (*r* = −0.064, *P* = 0.703); at 18 months: CRP (*r* = −0.106, *P* = 0.623), ESR (*r* = −0.079, *P* = 0.706). Similar to EULAR response, there was a negative weak correlation between the statin-exposed status and inflammatory markers values, with no statistical significance. According to Salkin scale, this is a very weak correlation.

## 4. Discussions

Even if statin's inhibiting effect on rituximab in RA patients is a highly discussed hypothesis, there are no significant studies showing this for a longer period of treatment than 6 months.

In our study, we have not demonstrated any influence of statins on the antirheumatic effect of rituximab in RA patients using EULAR response at 6 months and 18 months as outcome. Our results are similar to a previous study [12] regarding EULAR response at 6 months. Emery et al. showed that there is no evidence of less complete B-cell or plasmablast depletion in patients receiving a statin. Still, we found a very weak negative correlation between statin administration and EULAR response at 6 months (*r* = −0.073, *P* = 0.661) and 18 months (*r* = −0.197, *P* = 0.244). Perhaps this could be due to the relatively small size of the sample. To exclude this hypothesis, our study is still ongoing and we intend to widen the study cohort.

Although not statistically significant, there was also a negative correlation between the use of statin and values of inflammatory markers as ESR and CRP after 6 and 18 months of treatment with rituximab, similar to the one regarding the EULAR response. With a wider cohort, this tendency may be statistically proven.

These negative correlations could mean that the use of statin in RA patients receiving rituximab may inhibit the anti-inflammatory and antirheumatic effect of the anti-CD20 agent, thus decreasing the clinical and biological response and at the same time increasing the CV risk. Further studies with larger cohorts of patients should assess this hypothesis.

The clear relationship between RA disease activity and CV risk underlines the importance of a tight disease control. Given the fact that CV risk is considerably higher in RA patients and that statins may influence the clinical and inflammatory response in those receiving rituximab, we assessed the effect of statin on CV risk measured by mSCORE. There was a very weak correlation with the use of statin at baseline (*r* = 0.133, *P* = 0.413) and after 18 months (*r* = 0.191, *P* = 0.239). Furthermore, mSCORE was similar for both groups at baseline and after 18 months. These results lead to the conclusion that the use of statin did not improve the CV risk for the patients included in the study.

There are reports showing that patients with low/moderate CV risk according to SCORE suffered important CV complications [13]. Therefore additional tools for identifying patients with high CV risk are needed.

## 5. Conclusions

The treatment goal in RA is to achieve remission or low disease activity as soon as possible. Inflammatory burden increases CV risk and appropriate measures have to be taken. Long-term significant inhibitory effects of statins on rituximab treatment in (rheumatoid arthritis) RA have not been proved using clinical response scores, as well as biologic markers.

## Figures and Tables

**Table 1:** Patient characteristics at baseline.

Patient characteristic at baseline	Exposed (*n* = 17)	Unexposed (*n* = 24)	*P* value (2 tailed)
Age (years)	48.76 ± 10.57	38.79 ± 14.09	0.018
Women, *n* (%)	14 (82.35)	20 (83.33)	0.937
Previous biologic DMARDs* (*n*)	1.29 ± 0.588	1.25 ± 0.532	0.803
DAS28	5.16 ± 1.15	5.16 ± 1.44	0.993
CRP (mg/L)	18.52 ± 23.82	36.2 ± 45.26	0.172
ESR (mm/h)	36.44 ± 20.46	49.75 ± 27.71	0.109
RF (U/L)	87.21 ± 66.37	87.13 ± 65.71	0.997
Total serum cholesterol (mg/dL)	225.94 ± 58.343	181.83 ± 47.023	0.011
mSCORE	2.90 ± 3.54	3.27 ± 8.99	0.789

Values are mean ± SD.

*Number of biologic DMARDs prescribed prior to rituximab.

DMARD: disease-modifying antirheumatic drug; CRP: C-reactive protein; ESR: erythrocyte sedimentation rate; RF: rheumatoid factor; mSCORE: modified Systematic Coronary Risk Evaluation.
